# Activity of Specialized Biomolecules against Gram-Positive and Gram-Negative Bacteria

**DOI:** 10.3390/antibiotics9060314

**Published:** 2020-06-09

**Authors:** Tânia D. Tavares, Joana C. Antunes, Jorge Padrão, Ana I. Ribeiro, Andrea Zille, M. Teresa P. Amorim, Fernando Ferreira, Helena P. Felgueiras

**Affiliations:** Centre for Textile Science and Technology (2C2T), Department of Textile Engineering, University of Minho, Campus of Azurém, 4800-058 Guimarães, Portugal; taniatav@2c2t.uminho.pt (T.D.T.); joana.antunes@2c2t.uminho.pt (J.C.A.); padraoj@2c2t.uminho.pt (J.P.); afr@2c2t.uminho.pt (A.I.R.); azille@2c2t.uminho.pt (A.Z.); mtamorim@det.uminho.pt (M.T.P.A.); fnunes@det.uminho.pt (F.F.)

**Keywords:** antimicrobial peptides, essential oils, minimum inhibitory concentration, bactericidal, nosocomial

## Abstract

The increased resistance of bacteria against conventional pharmaceutical solutions, the antibiotics, has raised serious health concerns. This has stimulated interest in the development of bio-based therapeutics with limited resistance, namely, essential oils (EOs) or antimicrobial peptides (AMPs). This study envisaged the evaluation of the antimicrobial efficacy of selected biomolecules, namely LL37, pexiganan, tea tree oil (TTO), cinnamon leaf oil (CLO) and niaouli oil (NO), against four bacteria commonly associated to nosocomial infections: *Staphylococcus aureus*, *Staphylococcus epidermidis*, *Escherichia coli* and *Pseudomonas aeruginosa*. The antibiotic vancomycin and silver nanoparticles (AgNPs) were used as control compounds for comparison purposes. The biomolecules were initially screened for their antibacterial efficacy using the agar-diffusion test, followed by the determination of minimal inhibitory concentrations (MICs), kill-time kinetics and the evaluation of the cell morphology upon 24 h exposure. All agents were effective against the selected bacteria. Interestingly, the AgNPs required a higher concentration (4000–1250 μg/mL) to induce the same effects as the AMPs (500–7.8 μg/mL) or EOs (365.2–19.7 μg/mL). Pexiganan and CLO were the most effective biomolecules, requiring lower concentrations to kill both Gram-positive and Gram-negative bacteria (62.5–7.8 μg/mL and 39.3–19.7 μg/mL, respectively), within a short period of time (averaging 2 h 15 min for all bacteria). Most biomolecules apparently disrupted the bacteria membrane stability due to the observed cell morphology deformation and by effecting on the intracellular space. AMPs were observed to induce morphological deformations and cellular content release, while EOs were seen to split and completely envelope bacteria. Data unraveled more of the potential of these new biomolecules as replacements for the conventional antibiotics and allowed us to take a step forward in the understanding of their mechanisms of action against infection-related bacteria.

## 1. Introduction

Bacterial growth can be inhibited by antimicrobial agents, causing disruption of vital cellular functions resulting in rapid cell death. Typically, these agents act at the level of the bacterial membrane, which is a crucial structure for cell survival. The architecture and molecular components of the cell peripheral wall differ between Gram-positive and Gram-negative bacteria, particularly in what concerns membrane and cell wall structure and disposition [1,2]. The latter is more complex, containing two distinct lipid membranes, the cytoplasmic cell membrane and the outer membrane, with a thin layer of peptidoglycans in between. The outer membrane works as an additional compound-selective barrier [2]. It is highly permeable and contains lipopolysaccharides (LPS), the main lipid component, and a periplasmic space, where enzymes capable of degrading molecules introduced from the extracellular medium are present [1,2]. In Gram-positive bacteria, cytosol is enveloped by one bilayer membrane, the cytoplasmic membrane, attached to a thick layer of peptidoglycans, formed of linear polysaccharide chains cross-linked by short peptides that generate a three-dimensional (3D) rigid structure. In this case, lipoteichoic acids are adhered to the peptidoglycan layer. These components provide the bacterial membrane with an amphiphilic and anionic character [2,3,4].

Antimicrobial agents are generally lipophilic and cationic, allowing their positively charged side chains to bind to the negatively charged surface of the bacterial membranes. Subsequently, the lipophilic motifs interact with the lipid bacterial membrane, leading to instability and rupture of the membrane matrix and eventually to the cell death [1,5,6].

Currently, there is a vast array of antimicrobial biomolecules. For many years, the most widely used have been the antibiotics. However, their excessive consumption has led to an alarmingly high resistance development by bacterial pathogens, raising a serious global public-health problem [7,8]. Hence, the interest in the research for novel alternatives to antibiotics has been growing. Natural products are becoming very promising as antimicrobial agents, being considered safe and environmentally friendly [9,10]. Antimicrobial peptides (AMPs) of natural origin have been the focus of great interest as alternatives to conventional antibiotics. They play an important role in innate immunity, protecting the host against infections by microorganisms. AMPs are often cationic and amphiphilic molecules, with low molecular weight, and can be obtained from a variety of organisms (e.g., humans, insects, amphibian, bacteria, etc.) or synthesized as analogs of those naturally occurring [11,12,13]. LL37, for instance, is an AMP that belongs to the cathelicidin family, an important antimicrobial agent in humans. LL37 is essential for normal innate immune responses within infected and injured tissues, displaying a broad antimicrobial activity against both Gram-positive and Gram-negative bacteria. This AMP is also a promoter of tissue regeneration [14,15,16]. Pexiganan is an analog of the magainin peptides isolated from the skin of the African clawed frog. Like LL37, pexiganan is also reported to possess a broad spectrum of antibacterial action, displaying activity against over 3000 clinical isolates, including methicillin- and gentamicin-resistant *Staphylococcus aureus* [17,18]. On the other hand, essential oils (EOs) are composed of plant secondary metabolites that possess antibacterial, antiviral and antifungal activity, defending the host from microbiological invasion [19]. These consist of a complex mixture of chemical compounds, including terpenes, phenols, alcohols, aldehydes, ethers and ketones [20,21], most of which are hydrophobic or partially soluble in water. Usually, EOs have strong lipophilicity and volatility [9], making them suitable antibacterial agents for various applications encompassing anti-inflammatory and antioxidative properties. Nowadays, EOs application is found in many fields, including food preservation, cosmetics and biomedicine [22]. Cinnamon (*Cinnamomum zeylanicum*) is a well-studied EO with various biological properties, playing a key role in maintaining human health [23]. The cinnamon leaf oil (CLO) is mainly formed of eugenol, which is reported as the main compound responsible for its antimicrobial properties [24,25]. Like CLO, tea tree oil (TTO) is also known for its good antimicrobial properties. TTO can be obtained from *Melaleuca alternifolia*, an Australian native plant, and has been incorporated as an active ingredient in many topical formulations to treat cutaneous infections [26]. The main compound of TTO is terpinen-4-ol, being mainly responsible for its antimicrobial activity [26,27]. Niaouli oil is extracted from *Melaleuca viridiflora*, a perennial tree native to Australia, New Caledonia and the French Pacific Islands. This EO is of commercial importance due to its applications in aromatherapy and pharmaceutical preparations [28,29]. Its antimicrobial activity has also been established [30,31]. Although both AMPs and EOs have been reported as promising alternatives to antibiotics, particularly for their quick action and low tendency to induce resistance, there is still much to understand about their mechanisms of action against selected microorganisms.

The present study aimed to screen and further detail the antibacterial properties of both AMPs and EOs against four common nosocomial bacteria, *S. aureus*, *Staphylococcus epidermidis*, *Escherichia coli* and *Pseudomonas aeruginosa*, providing an original and critical overview on the differences and similarities of these relatively novel wide-spectrum bactericidal compounds. Indeed, the selected EOs produced from *Folha d’Água* Company from Portugal have never been examined in such light, nor have their antimicrobial performance or mechanisms of action been compared with AMPs in such detail. Their efficiency was accessed in light of their minimal inhibitory concentration (MIC) and kill-time kinetics, and also comprises a critical discussion of the exerted effect on the bacteria morphology, extrapolating from these findings their inherent mechanisms of action against the aforementioned microorganisms.

## 2. Materials and Methods

### 2.1. Materials

Commercial nanopowder of silver nanoparticles (AgNPs) coated with polyvinyl pyrrolidone (PVP), possessing 20–30 nm in diameter, were acquired from SkySpring Nanomaterials, Inc. (USA). The antibiotic vancomycin hydrochloride was purchased from Sigma (USA). The AMPs pexiganan (Mw 2477.17 Da, GIGKFLKKAKKFGKAFVKILKK) and LL37 cathelicidin (Mw 4493.3 Da, LLGDFFRKSKEKIGKEFKRIVQRIKDFLRNLVPRTES) were provided from Innovagen AB (Sweden) and IscaBiochemicals (UK), respectively. EOs TTO (ρ = 0.895 g/cm^3^, extracted from *M. alternifolia*), CLO (ρ = 1.049 g/cm^3^, extracted from *C. zeylanicum*) and NO (ρ = 0.913 g/cm^3^, extracted from *M. viridiflora*) were purchased from *Folha d’Água* Company (Portugal). Trypticase soy broth (TSB) and trypticase soy agar (TSA) were acquired from VWR (USA), while Mueller Hinton broth (MHB) was obtained from CondaLab (Spain). All tested bacteria were supplied from American Type Culture Collection (ATCC, Spain), encompassing Gram-positive bacteria, *S. aureus* (ATCC 6538) and *S. epidermidis* (ATCC 35984), and Gram-negative bacteria, *E. coli* (ATCC 25922) and *P. aeruginosa* (ATCC 25853).

### 2.2. Antimicrobial Solutions Preparation

Water dispersions of AgNPs were prepared at concentrations ranging from 5000 to 1.95 μg/mL, by sonification for 30 min in a Branson 3510 bath (355 W, 50–60 Hz), followed by another 30 min in an Optic Ivymen Sytem CY-500 tip (450 W, 20 Hz, 80%). Dispersion of NPs was assessed via scanning transmission electron microscopy (STEM), using an ultrahigh-resolution field emission gun scanning electron microscope (FEG-SEM, NOVA 200 Nano SEM, FEI Company). Secondary electron images were obtained at an electron accelerating voltage of 15 kV. The antibiotic vancomycin (2000–1.95 μg/mL) was prepared in distilled water (dH_2_O). LL37 (1000–0.98 μg/mL) was prepared in phosphate buffered saline solution (PBS, pH 7.4), while pexiganan was dissolved in dH_2_O at the same concentration range. EOs were diluted in MHB at concentrations from 500 to 0.18 μg/mL. Maximum and minimum concentrations were defined based on the literature for these or similar compounds.

Before contact with the bacteria suspensions, all solutions were vortexed for 30 s to guarantee the homogeneous distribution of the antimicrobial agents.

### 2.3. Agar-Well Diffusion Assay

The antibacterial activity of the AgNPs, antibiotic, AMPs and EOs was assessed against the four bacteria after a 24 h incubation period. 90 mm diameter Petri dishes were prepared with 15 mL TSA and left to solidify. Bacteria suspensions were diluted in TSB to a concentration of 2 × 10^6^ colony forming units (CFUs)/mL. Afterwards, 200 μL of each inoculum was uniformly spread on the solid plates. Sterilized punchers were used to generate 6 mm diameter holes on the agar. 40 μL of each antimicrobial agent, at the highest concentration studied, were introduced in the respective holes. Plates were incubated at 37 °C for 24 h. Thereafter, the zones of inhibition (ZoI) observed were measured to confirm the antimicrobial agents’ efficacy.

### 2.4. Minimum Inhibitory Concentrations (MICs)

MICs were determined using the broth microdilution procedure described by Wiegand et al. [32], which is an adaptation of the standard method published by the Clinical and Laboratory Standards Institute (CLSI) and the European Committee on Antimicrobial Susceptibility Testing (EUCAST) [33].

Working solutions were prepared for each antimicrobial agent, as described in Section 2.2, and added to the first column of 96-well plates in a volume of 100 μL. Serial dilutions (1:2) were made with MHB in the consecutive wells, to a final volume of 50 μL. Then, to each of these wells, 50 μL of the bacteria suspensions prepared at 2 × 10^7^ CFUs/mL in MHB were added. Agent-free bacteria suspensions and culture media were used as controls. Absorbance readings at a wavelength of 600 nm (EZ READ 2000 Microplate Reader, Biochrom, UK) were made before and after plate incubation for 24 h at 37 °C and 100 rpm. The MIC value for each agent/bacteria combination was established as the concentration at which bacteria did not show any growth, determined visually, and confirmed by the differences in absorbance readings. The number of viable cells at the MICs and the concentrations at its vicinity (concentration higher and lower than MIC value) was determined by estimating the number of CFU s/mL. Briefly, aliquots of 10 μL of each cell suspension, diluted from 10^1^ to 10^6^ in PBS, were cultured in TSA plates for 24 h at 37 °C, and colonies were counted.

### 2.5. Kill-Time Analysis: Bacteria Viability

Bacteria suspensions were prepared at 2 × 10^7^ CFUs/mL in MHB and combined at a 50% (*v*/*v*) with the antimicrobial agents at MICs. Control groups were prepared without the addition of any agent. Agent/bacteria solutions were incubated at 37 °C and 100 rpm. After 0 (before agent action), 1, 2, 4, 6, 10, 14, 18 and 24 h of incubation, bacteria were serially diluted (10^1^ to 10^6^ in PBS), cultured on TSA plates and further incubated for another 24 h at 37 °C. Colonies of surviving bacteria were counted and reported as mean ± standard deviation (SD). Data was collected in triplicate and processed using the GraphPad Prism 7.0 software.

### 2.6. Cell Wall Disruption: Scanning Electron Microscopy (SEM) Observations

To have an overview of the antimicrobial agents’ capacity to interfere with the cell morphology of Gram-positive and Gram-negative bacteria, visual studies resorting to SEM were conducted. Bacteria suspensions were prepared at 2 × 10^7^ CFUs/mL in MHB and combined at a 50% (*v*/*v*) with AgNPs, antibiotic, AMPs and EOs at half of the concentration of the MIC value, in this way, allowing for live and dead cells to be observed simultaneously and differences in morphology be identified more easily. 500 μL of each solution (250 μL agent + 250 μL bacteria) were left in direct contact with 12-well tissue culture plates (TCPS) and incubated at 37 °C for 24 h at 100 rpm. Afterwards, culture media was removed and 500 μL of 2.5% (*v*/*v*) glutaraldehyde in PBS were added to each sample for 1 h at room temperature, to promote cell fixation to the TCPS wells. Plates were gently rinsed with dH_2_O and submitted to a dehydration process using serial ethanol dilutions of 55%, 70%, 80%, 90%, 95% and 100% (*v*/*v*), and each solution was left in the TCPS for 30 min at room temperature, and then carefully discarded. After the last solution, the remaining ethanol was evaporated at room temperature [34]. Discs were cut from each TCPS well, containing the fixated and dehydrated bacteria, using a hot press-on apparatus and covered with a thin film (10 nm) of Au-Pd (80–20 wt%) in a 208HR high-resolution sputter coater (Cressington Company, Watford WD19 4BX, United Kingdom) coupled to a MTM-20 Cressington high-resolution thickness controller. Bacteria cells’ morphology was observed via FEG-SEM (NOVA 200 Nano SEM, FEI Company), using an electron accelerating voltage of 10 kV.

## 3. Results and Discussion

### 3.1. Agar-Well Diffusion

**Figure 1:**
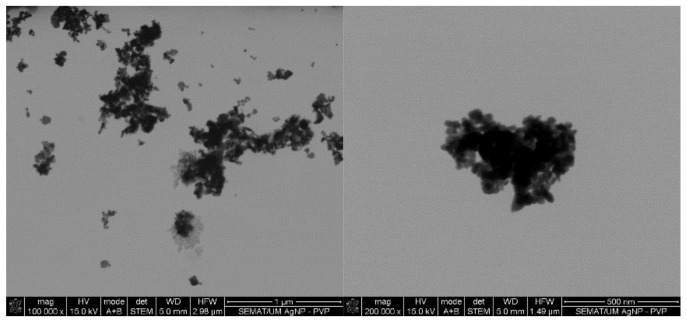
Silver nanoparticles (AgNPs) colloidal dispersion scanning transmission electron microscopy (STEM) micrographs at magnifications of ×100,000 and ×200,000 with evidence of formation of NPs clusters.

Initial screening of the antimicrobial activity of the investigated agents was studied against the four microorganisms using the agar-well diffusion test, which showed the presence and absence of ZoI (Table 1). All agents revealed different degrees of antibacterial activity against the tested bacteria. The antibacterial action was classified following the Rota et al. scale, which reports weak activity with a ZoI (halo) ≤ 12 mm, moderate activity with a ZoI ranging between >12 and <20 mm, and strong activity with a ZoI zone ≥ 20 mm [35,36]. AgNPs display a low-activity ZoI, particularly against Gram-negative bacteria. It has been reported that metal nanoparticles tend to form agglomerates when in colloidal dispersions, which reduces their diffusivity, limiting the contact with the bacteria [37]. Here, the presence of agglomerates is evident (Figure 1), supporting this statement. The agar tortuosity may also influence this phenomenon by hindering the AgNPs permeation through the culture media. Vancomycin antibiotic action occurs at the bacterial cell wall through the disruption of the synthesis of its major constituent, peptidoglycan. Vancomycin binds to the terminal carboxyl group of D-alanyl-D-alanine within nascent peptidoglycans of the cell wall, preventing the formation of lipid II, a key “shuttle carrier” of the peptidoglycan monomers [38,39]. This molecule forms a series of hydrogen bonds with the peptide backbone blocking its processing [40,41,42]. It is true that Gram-negative bacteria outer cell wall is fairly impermeable to large glycopeptide molecules such as vancomycin [2]. Therefore, vancomycin was reported as weak against the Gram-negative bacteria but strong against the Gram-positive bacteria. As such, the absence of the outer membrane and periplasm in the Gram-positive bacteria allows a higher permeability of vancomycin through its cellular wall, which possess a hydrophilic porous structure [43,44,45].

AMPs LL37 and pexiganan are well-known effective antimicrobial agents. However, data from Table 1 revealed a weak to moderate ZoI against the selected bacteria, respectively. The action of LL37 is suspected to have been compromised by the presence of salts within the solvent, PBS (recommended by the manufacturer), as the LL37 structure varies with the ionic charge of the solvents, possibly reducing its agar diffusion capacity [46]. On its turn, pexiganan action was considerably superior to that of LL37, particularly against *S. epidermidis* and *P. aeruginosa*. These findings are consistent with the literature [18]. 

The EOs displayed a variable degree of antibacterial activity against the tested bacteria. Interestingly, they were all found to be more effective against the *S. aureus* bacteria, with a moderate (NO) to strong (TTO and CLO) activity. These results were closely followed by the *S. epidermidis* and *E. coli* bacteria. *P. aeruginosa* was the least susceptible to the EOs action, with weak (NO) to moderate (TTO and CLO) ZoIs being formed. Once again, this can be explained by the differences in cell wall structure between Gram-positive and Gram-negative bacteria, with the latter being capable of restricting diffusion of hydrophobic compounds through its LPS envelope [36]. From the three examined oils, NO was the least effective (≈1.6-fold lower than the remainder oils). The EOs antimicrobial activity is attributed to the presence of several low molecular weight phenols, terpenes and aldoketones within their composition [47]. Hence, the higher ZoI formed by TTO or CLO could be explained by the presence of volatile compounds. TTO encompasses terpinen-4-ol (40%), γ-terpinene (20%), α-terpinene (10%), 1,8-cineole (3%), α-pinene (3%) and limonene (1%) (*v*/*v*). TTO comprises in its formulation eugenol (80%), β-caryophyllene (4%), benzyl benzoate (4%), cinnamaldehyde (3%), linalool (2%) and α-terpinene (1%) (*v*/*v*). All these compounds are known to contribute significantly for the EOs antimicrobial activity [26,47,48]. NO is known to possess terpinen-4-ol, γ-terpinene, α-terpinene, 1,8-cineole, α-pinene, limonene, β-caryophyllene and linalool, but with a major contribution of 1,8-cineole (60%) (*v*/*v*) to its antimicrobial activity. 1,8-cineole is known to exert lower antimicrobial action than terpinen-4-ol or eugenol [28,49,50,51,52].

### 3.2. MICs

The obtained MICs of the selected antimicrobial agents for each bacterium are shown in Table 2. MICs evaluation showed that the selected antimicrobial agents were active against all tested bacteria, which fairly agrees with the data obtained from the agar-well diffusion studies

From the entire list of tested agents, the MICs of the AgNPs were the highest (4000–1250 μg/mL). The main mechanism of action of AgNPs against bacteria requires the attachment and interaction of multiple NPs to the cell surface [53]. This induces the disruption of the bacteria membrane functions and dissipation of the proton motive force. Due to their high surface-to-volume ratio, small AgNPs of few nanometers may even alter the morphology of the cell wall, increasing their diffusion towards the intracellular space, ultimately leading to the cell death [54]. Here, even though PVP was used as a dispersant agent to produce AgNPs, there was still a large tendency to form agglomerates in colloidal dispersions (Figure 1). Consequently, a large number of NPs were expected to be attracted and immobilized along the membrane of each bacterium to induce a bactericidal effect. Between the tested organisms, *P. aeruginosa* was the most susceptible to the AgNPs action. It has been postulated that Gram-negative bacteria are more susceptible to AgNPs because AgNPs positive charges interact with the outer LPS membrane with more affinity than with the Gram-positive cell wall, which is thought to have fewer interaction sites [55]. This effect, however, was not verified on the *E. coli*, which MIC was equal to *S. aureus* and *S. epidermidis*. 

As expected, vancomycin was more effective against Gram-positive than Gram-negative bacteria, with MICs being 120-fold lower [2]. The pexiganan MICs were also consistent with the ZoI findings, being in a range close to that reported in the literature [17,18]. On the contrary, the LL37 action was found to be superior against Gram-negative bacteria, even though the ZoIs were more evident against the Gram-positive ones (Table 1). Again, these results imply the difficulty of the peptide in diffusing through the solid media [46]. Regarding the tested bacteria, the LL37 ability to act more effectively against the *E. coli* and *P. aeruginosa* bacteria is related to its electrostatic interaction and its structure. It has been reported that LL37s first interaction step is promoted by its electrostatic attraction to lipid A and to phosphate groups linked to sugar residues of LPS [56]. Subsequently, α-helix peptides, such as LL37, generally act via a membranolytic mechanism. The helix formation allows an optimal spatial arrangement of the aliphatic side chains for membrane insertion. Strong hydrophobic interactions are formed between these chains and the lipid layer of Gram-negative bacteria, stabilizing the AMP helical conformation, thus reducing main-chain hydrophobicity and allowing for a deeper and easier insertion into the bilayer [57,58].

As shown in Table 2, the EOs displayed variable levels of MICs for each tested microorganism. CLO had the lowest MICs (19.7–39.3 μg/mL) from the group, while NO had the highest (137.0–365.2 μg/mL). These findings corroborate the ZoI examinations (Table 1). The EOs differences in chemical composition and presence of more effective low molecular weight antibacterial compounds on CLO than on NO, exerts a major role in their antibacterial activity efficacy [47,48,49]. To the best of the authors’ knowledge, NO has not yet been tested against these specific strains. Still, in other cases, MICs have been reported around 300 and 500 μg/mL [28]. Regarding the TTO MICs, even though they are slightly superior to those reported in the literature, the pattern of efficiency remains: *P. aeruginosa* < *S. epidermidis* < *S. aureus* < *E. coli* [26].

### 3.3. Kill-Time Analysis

**Figure 2:**
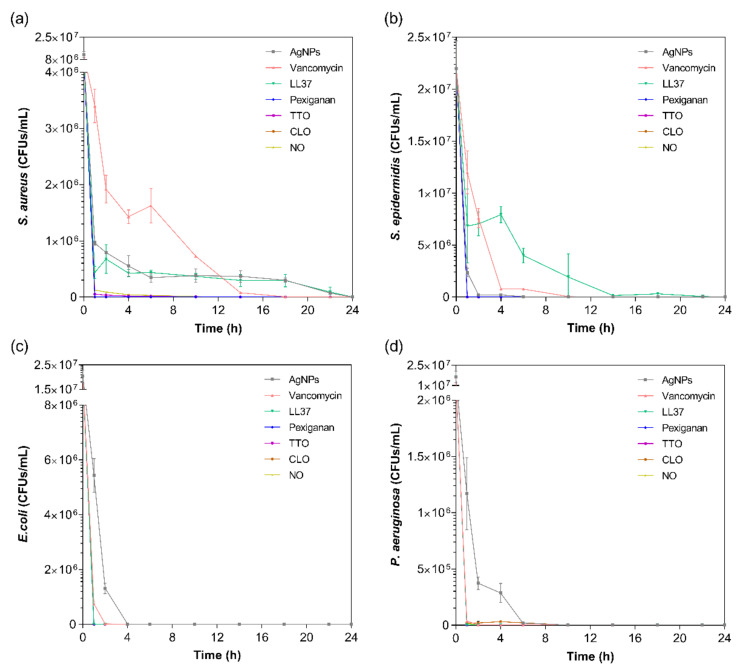
Kill-time curves of AgNPs, vancomycin, LL37, pexiganan, TTO, CLO and NO, at the MICs concentrations, against (**a**) *S. aureus*, (**b**) *S. epidermidis*, (**c**) *E. coli* and (**d**) *P. aeruginosa*, up to 24 h culture. Data derived from three repetitions. Positive controls for each bacterium (growth without agent) were also conducted, reaching maximum values of ≈8.0 × 10^8^ colony forming units (CFUs)/mL for *S. aureus*, ≈1.6 × 10^9^ CFUs/mL for *S. epidermidis*, ≈1.4 × 10^9^ CFUs/mL for *E. coli* and ≈8.7 × 10^8^ CFUs/mL for *P. aeruginosa* after 24 h culture (data not shown).

The kill-time kinetics for each agent was determined by the number of remaining viable cells at specific time points, namely 1, 2, 4, 6, 10, 14, 18, 22 and 24 h (Figure 2). Although MIC values are commonly used to predict the antibacterial action of any agent, such data does not consider the exposure and action time of the agent against each bacterium. As such, the kill-time kinetics was used to unravel the antibacterial potency of the tested compounds over time.

For all bacteria/agent combinations, bactericidal action was observed from the first hour of contact. In fact, the action of the pexiganan, TTO, CLO and NO was so immediate that after 2 h of contact, very little bacteria remained (≈3 × 10^4^ CFUs/mL of *S. aureus* with TTO, ≈2 × 10^4^ CFUs/mL of *P. aeruginosa* with CLO, and ≈3 × 10^4^ CFUs/mL of *S. aureus* with NO; the remainder were all killed at this point). The main bactericidal action of the AMP pexiganan results from irreversible membrane-disruptive damage, which based on the mechanism of action of magainin (precursor), is expected to exert its antimicrobial action very quickly, within the first moments of interaction [59]. Our data is consistent with this information and with previous reports [17]. In fact, all bacteria were dead after 1 h of contact, with no regrowth being observed within the 24 h tested. Regarding the EOs, the susceptibility pattern for each bacterium did not appear to predict the activity of the EO. For instance, even though TTO required a very small concentration to kill *S. aureus*, it took 6 h for this bacterium to be eliminated, whereas TTO only required 1 h to eradicate the other bacteria. The same occurred with NO. On its turn, CLO followed the pattern of MIC concentrations, requiring 6 h to kill the *P. aeruginosa* and less than 1 h for the other bacteria. The EOs mechanism of action relies on their inherent hydrophobicity, which enables them to accumulate in the cell membrane, disturbing its structure and functionality, and causing an increase of permeability [36,60]. Despite sharing a similar membrane and cell wall structure and disposition, it is known that *E. coli* and *P. aeruginosa* possess distinct lipid and protein composition and concentration [61]. This most likely is the main factor for the observed MIC differences between these bacteria. Even though it is not yet clear at which stage of bacteria development the EOs are the most effective, it is generally accepted that they stimulate cell autolysis in exponential and stationary cell phases [62]. Our findings demonstrate that exponentially growing cells are very susceptible to the EOs’ action. 

In case of the LL37, the action was quicker against Gram-negative bacteria compared to Gram-positive ones. It has been shown that permeabilization of the cytoplasmic membrane of Gram-positive bacteria by LL37 is considerably slower than against Gram-negative [57,58]. Interestingly, vancomycin also had a faster action against Gram-negative bacteria than against Gram-positive, even though all available data up until now has revealed its higher effectiveness towards the former. This may have happened due to the differences in MIC values. While *S. aureus’* and *S. epidermidis’* MIC was only 7.8 μg/mL, 1000 μg/mL of the antibiotic was necessary to kill *E. coli* and *P. aeruginosa*. Several studies have shown that the bactericidal action, namely kill-time kinetics, of a given antimicrobial agent is dependent on the concentration to which the microorganisms are exposed [17,63]. For the four bacteria, AgNPs was the agent that took the longest time to eliminate the entirety of CFUs. As seen in Figure 1, AgNPs clusters prevented its homogenous dispersion within the solution, which implies that AgNPs were not evenly available to bind to sites at the bacteria membrane. As AgNPs are only capable of disrupting the bacteria cell membrane after proper binding, subsequently infiltrating the cytosol to induce cell death [54], this limited distribution may have required additional time than the free-state, non-clustered molecules, characteristic of the other tested agents.

### 3.4. Cell-Wall Disruption: Mechanisms of Action

**Figure 3:**
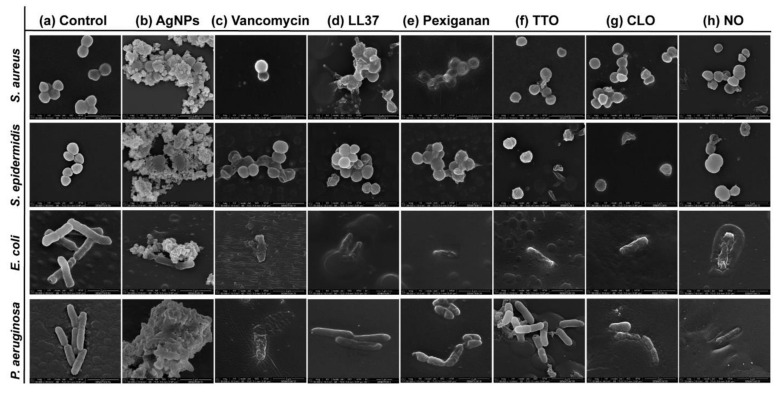
Micrographs of *S. aureus* (Sa), *S. epidermidis* (Se), *E. coli* (Ec) and *P. aeruginosa* (Pa) bacteria untreated (control) and treated with the selected antibacterial agents, at the smallest tested concentration just before establishing the MIC value. Concentrations were defined at 2000 μg/mL in Sa, Se and Ec and 625 μg/mL in Pa for AgNPs; 3.9 μg/mL in Sa and Se and 500 μg/mL in Ec and Pa for vancomycin; 250 μg/mL in Sa and Se, 62.5 μg/mL in Ec and 125 μg/mL in Pa for LL37; 15.7 μg/mL in Sa and Pa, 3.9 μg/mL in Se and 31.3 μg/mL in Pa for pexiganan; 33.6 μg/mL in Sa, 89.5 μg/mL in Se, 16.8 μg/mL in Ec and 134.3 μg/mL in Pa for TTO; 15.7 μg/mL in Sa and Se, 9.9 μg/mL in Ec and 19.7 μg/mL in Pa for CLO, and 68.5 μg/mL in Sa and Ec, 91.3 μg/mL in Se and 182.6 μg/mL in Pa for NO. These concentrations allowed for live and dead cells to be observed simultaneously, with morphological differences being more easily identified.

Possible mechanisms of membrane interaction and disruption of the tested bacteria have been observed via SEM imaging through exposition to the selected agents at half of the MICs concentrations, for 24 h. Figure 3 shows the morphology of the bacteria with and without contact with AgNPs, vancomycin and selected AMPs and EOs. As expected, the control (without agent) of the *S. aureus* and *S. epidermidis* bacteria revealed a coccoid-shaped conformation with a smooth and uninterrupted surface. Both cell types tended to be arranged in grape-like clusters and cell propagation was recurrently observed [64,65]. The morphology of the *E. coli* and *P. aeruginosa* bacteria was also very similar, with both displaying a rod-shaped architecture. *E. coli* cells are typically 2.0 μm long and 0.25–1.0 μm in diameter. *P. aeruginosa* cells present similar dimensions and, in many cases, polar flagella, which endows the bacteria with motility, may also be evident [66,67,68]. Here, however, that was not the case. Gram-negative control cells presented an even distribution, displaying multiple cells undergoing polar binary fission.

There are two mechanisms of action widely accepted for AgNPs, the contact killing and the ion-mediated killing. Contact killing is clearly evidenced in all tested microorganisms (Figure 3b). The positively charged AgNPs are attracted to the negatively charged bacteria surface, enabling NP attachment along the cell surface. This action induces physical changes in the bacterial membrane, compromising its integrity and inducing the diffusion of NPs towards the intracellular space. Here, AgNPs species, such as Ag^+^ ions, are released and interact effectively with specific functional groups in proteins, consequently inhibiting intracellular metabolic functions and causing protein denaturation. At this point, the cellular content will leak, ultimately leading to the cell death [69,70]. This effect is particularly evident against the *P. aeruginosa* bacteria, as the rod-shaped morphology is barely evident in most cells and the cell content is already fused with the AgNPs clusters. Additionally, AgNPs are also capable of producing high levels of reactive oxygen species (ROS) and free radical species that may interact electrostatically with the cell wall, generating a charge superior to its tensile strength, therefore also compromising its integrity [71]. 

Direct inhibition of the Atl amidase domain (major domain in staphylococcus bacteria cell wall), due to vancomycin-induced inhibition of cell wall synthesis, causes defects in the cell morphology and alters cell membrane permeability (*S. aureus* in Figure 3c), ultimately leading to autolysin-triggered cell rupture, release of cell content and death (*S. epidermidis* in Figure 3c) [40,41,42]. Even though the mode of action against *S. aureus* and *S. epidermidis* is similar, Figure 3c recorded the alterations induced by vancomycin at two stages, an earlier for *S. aureus* and a more advanced for *S. epidermidis*. This occurs because vancomycin requires more time to kill the first bacteria than the second (Figure 2), thus allowing for the extrusion and reduction of cell content to occur more intensively in the *S. epidermidis* upon 24 h exposure. The same explanation can be applied to the Gram-negative bacteria. The large size of this glycopeptide precludes it from being capable of penetrating the outer membrane of Gram-negative bacteria and inducing morphology changes and autolysin-triggered cell rupture, as happens in Gram-positive bacteria [43,44,45]. We postulate that because of the superior concentration of vancomycin (1000 μg/mL) required to kill these bacteria and its fast action, these molecules accumulate along the surface of the bacterium, isolating it from the media and respective nutrients, and providing enough steric hindrance to prevent peptidoglycan synthesis. Hence, starving the bacteria may trigger a set of events somewhat similar to those characteristics of the Gram-positive bacteria that culminate in cell rupture, release of cell content and death. As the kill-time kinetics is so fast, after 24 h exposure, it was only possible to capture fragments of individual cell membranes and residues of cell content for *E. coli* and a very advanced deformed morphology for *P. aeruginosa*.

AMPs are unique biomolecules which mode of action may be divided into direct killing (membrane and non-membrane target) and immune modulation. LL37-treated *S. aureus* displayed clear irregular protruding structures, to an extent that at least some bacteria appeared to have extruded cytoplasm and become embedded by exudate. In the case of *S. epidermidis*, morphological changes were easily distinguished, with a small leakage of cytoplasm content also being perceived within the bacteria cluster. LL37 performs its bactericidal action by electrostatic binding of its cationic molecules to the outer surface of the bacterial cell. This peptide uses the carpet-like mechanism, in which the AMP coat the microbial membrane up to saturation, after which point wormholes are formed, or the toroidal mechanism of action, in which after binding to the phospholipid head groups, the AMP aligns and inserts into the membrane and cluster into unstructured bundles that span the membrane and generate channels from each of the intracellular content leaks [12,14]. LL37 is amphiphilic in nature with hydrophobic and hydrophilic residues aligned on opposite sides of the peptide. This facilitates their penetration through the cell membrane [72], which results in inhibition of nucleic acid and protein biosynthesis, followed by leakage of the cell cytoplasm into the extracellular space, causing bacteria death [73]. Although the action of LL37 against Gram-negative bacteria is very similar to that described against Gram-positive bacteria, the rate at which cytoplasmic permeabilization occurs is superior. The peptide α-helix structure forms strong hydrophobic interactions with the outer membrane and its LPS and O-antigen layers of the Gram-negative bacteria, which quickly saturates, thus allowing for a deeper and faster insertion into the bilayer [57,58]. The halting of growth occurs shortly after the translocation of LL37 across the outer membrane into the periplasmic space, and is followed by the rapid interference with the synthesis of the nascent curved cell envelope (the outer membrane, cytoplasmic membrane, peptidoglycan layer and LPS layer) and its intracellular organelles [74]. These phenomena may explain the more advanced state of deformation/decomposition registered by the *E. coli* and *P. aeruginosa* bacteria after 24 h exposure to the LL37 (Figure 3d). Here, a substantial decrease in signal intensity correlates to cells being depleted of intracellular organelles, in a bed of organic matter (very easily identified for *E. coli*). Just as LL37, pexiganan also exert its antibacterial effect by forming toroidal pores in the bacterial membrane [75]. The cationic AMP with divalent cation binding sites disrupts the arrangement of the hydrophobic and hydrophilic sections of the bilayer, inducing a local curvature which alters the morphological appearance of the cell (evident in all bacteria from Figure 3e). As the pores are transient upon disintegration, pexiganan can translocate to the inner cytoplasmic leaflet entering the cytoplasm and potentially targeting intracellular components [17,76]. As the antimicrobial action of AMPs, including pexiganan, is related to its availability, bacteria exposed to a higher concentration of pexiganan were more prone to disintegrate and release their cellular content during the 24 h contact. This was particularly clear on the *E. coli*, for which MIC was the highest (62.5 μg/mL). As observed, AMP kills bacteria very quickly, within the first 2 h of exposure (Figure 2), by physically disrupting the cell membrane, which is a highly conserved structure, thus the development of resistance may not be an immediate concern, which potentiates further research into its clinical application [77,78]. In fact, all mutagenesis attempts to induce pexiganan resistance in *E. coli* and *S. aureus* failed [79].

It is generally accepted that EOs act primarily against the cell cytoplasmic membrane of the microorganism. Their inherent hydrophobicity is an important characteristic that enables them to accumulate within the cell membrane, disturbing its structure and functionality, and causing an increase of permeability. Leakage of intracellular components and impairment of microbial functions can then occur, ultimately causing cell death [36,60]. Even though in *S. aureus* the action of TTO appeared to have only compromised the cell wall with little cell content being released, in *S. epidermidis*, its effect is very pronounced with the complete disintegration of the cell membrane and substantial leakage to the extracellular space. Here, cell lysis is clear. This difference in behavior between staphylococcus bacteria may be correlated with the kill-time kinetics of the EO. While *S. aureus* withstands viable cells for 6 h, the other tested microorganisms were all eliminated within 1 h. In fact, disintegration of the cell wall, leakage of intracellular components and cell lysis are especially noticeable in *E. coli* and *P. aeruginosa* (Figure 3f). Another explanation relies on the TTO action mode against *S. aureus*. Data suggests that the primary mechanism of action against this bacterium may not be just gross cell wall damage, as it happens with the other bacteria, but rather a combination of the weakening of the cell wall and subsequent rupture of the cytoplasmic membrane due to osmotic pressure with the impairment of microbial autolytic enzyme systems, which eventually induce a delayed death [50,51]. Obvious detrimental effects on the cell membrane morphology were also shown when bacteria were treated with CLO and NO. Microstructural observations demonstrated these EOs’ capacity to increase cell permeability, distorting the cell membrane integrity and generating holes or wrinkles. The latter were particularly clear on the Gram-negative bacteria, possibly due to their outer cell membrane and thin peptidoglycan wall. An incomplete and deformed shape was observed in some *S. aureus* and *S. epidermidis* cells. Cell shrinkage and blebbing-like architectures were also detected among these microorganisms. Interestingly, intracellular leakage was only observed on *S. aureus*, even though rupture and lysis of membranes with a “breaking-in-half”-like deformation was predominant in *S. epidermidis*. CLO is composed of 80% eugenol, and its antibacterial action can be attributed to a double bond in the α,β positions of the side chain and a methyl group located in the γ position. Typically, eugenol exhibits higher activity against Gram-negative bacteria than Gram-positive bacteria [50]. Deformation of the bacterial cell wall of Gram-negative bacteria is evident upon exposure to CLO. It appears that the EO surrounds the cells, isolating them, for an effective permeabilization of the membrane. Indeed, since these bacteria are relatively more resistant to hydrophobic biomolecules, to overcome their impermeability, EOs rely on the organisms isolation to slowly traverse through the outer wall porins [50,80]. This phenomenon is also evident on the Gram-negative bacteria treated with NO. Still, the differences in concentration between CLO and NO necessary to accomplish such a task (Table 2) may be accompanied by a different mechanism of action against these bacteria. As the concentration of phenolic compounds in NO is smaller than on CLO [47,48,49], the first may rely on the interference with enzymes involved in the production of energy to induce cell lysis, while the second may denature proteins present at the intracellular space.

## 4. Conclusions

The wide-spectrum antibacterial efficacy of AMPs and EOs was further thoroughly analyzed in this work. The tested AMPs, LL37 and pexiganan displayed efficacy against the tested bacteria. Nevertheless, in comparison to LL37, pexiganan exhibited a considerably lower MIC (ranging between 2- and 64-fold lower), in addition to its impressive kill-time against all bacteria (≤1 h), whereas LL37 only exhibited such killing rate against Gram-negative bacteria. Interestingly, the LL37 antimicrobial action was apparently hindered by the agar tortuosity, thus displaying a limitation in its application. The most effective EO was CLO, exhibiting a lower MIC than TTO (between 1.7- and 6.8-fold) and NO (between 5.2- and 9.3-fold) and a fast kill-time, 1 h for Gram-positive bacteria and *E. coli*, and 6 h for *P. aeruginosa*. The main target of most of the tested agents was cell envelope, highlighting both their wide-spectrum potential and that they are not prone to rapidly induce bacterial resistance. These properties make them highly valuable biomolecules for the urgent “biocide transition” to reduce the need and use of non-effective conventional antibiotics. This is a first step in a larger investigation in which the competitive and synergistic behavior of these biomolecules and their affinity towards biodegradable fibrous constructs will be the envisaged goals. Research will soon be published on these subjects.

## Figures and Tables

**Table 1:** Zones of inhibition (ZoI) of selected antimicrobial agents against Gram-positive and Gram-negative bacteria (*n* = 3, mean ± standard deviation (SD)). The diameter of the holes (Ø = 6 mm) was included. Images were collected without regard for size proportionality, only being used as representations of the halos formed.

Antimicrobial Agents	ZoI Diameter (mm)			
	*S. aureus*	*S. epidermidis*	*E. coli*	*P. aeruginosa*
**AgNPs**	11.5 ± 1.7 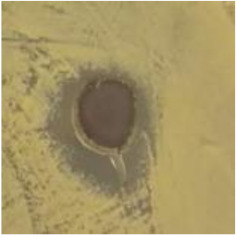	10.6 ± 0.6 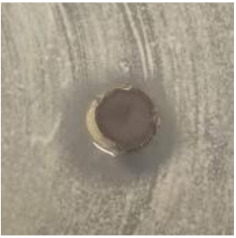	8.8 ± 0.5 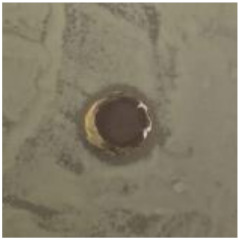	8.8 ± 3.0 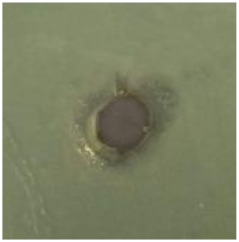
**Vancomycin**	22.5 ± 0.5 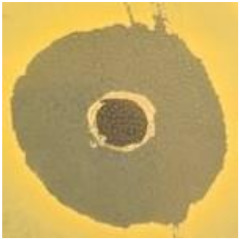	22.5 ± 0.5 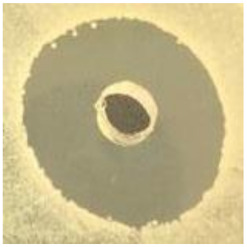	8.0 ± 0.1 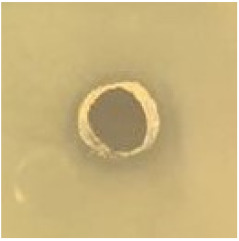	8.0 ± 0.2 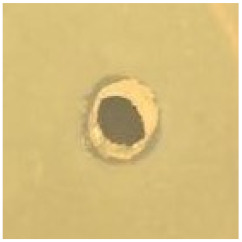
**LL37**	6.5 ± 0.1 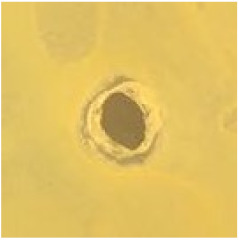	6.5 ± 0.5 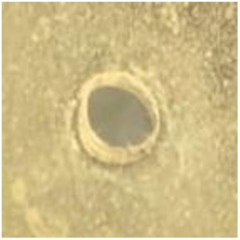	6.3 ± 0.1 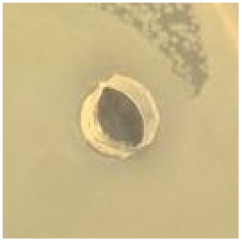	6.2 ± 0.1 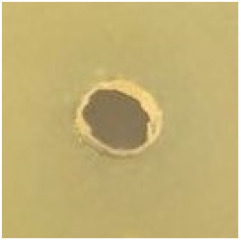
**Pexiganan**	9.0 ± 0.5 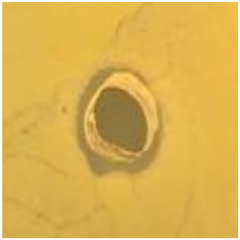	12.2 ± 0.6 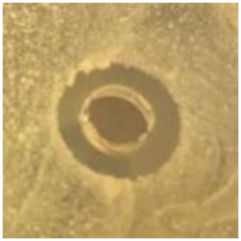	8.0 ± 1.5 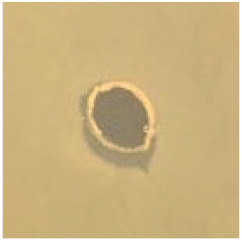	12.0 ± 0.1 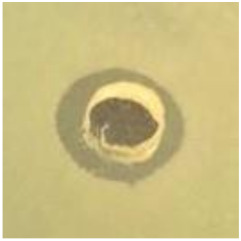
**TTO**	20.2 ± 0.1 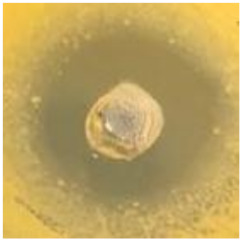	15.0 ± 0.5 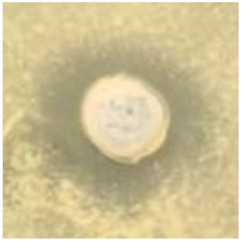	15.5 ± 0.5 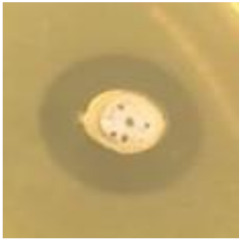	13.3 ± 0.3 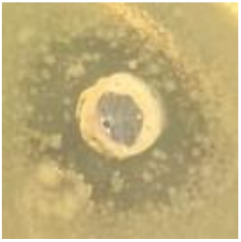
**CLO**	21.5 ± 0.5 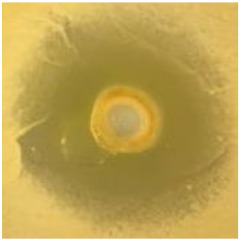	15.0 ± 1.0 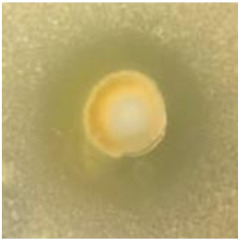	15.0 ± 1.9 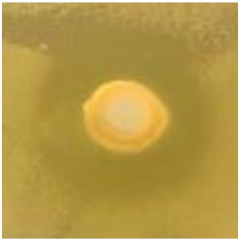	15.0 ± 0.6 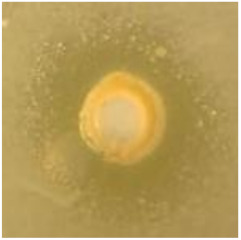
**NO**	14.7 ± 0.4 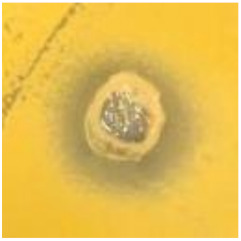	10.0 ± 0.5 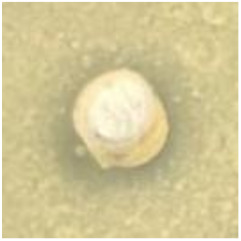	11.5 ± 0.5 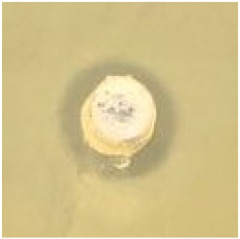	6.8 ± 0.5 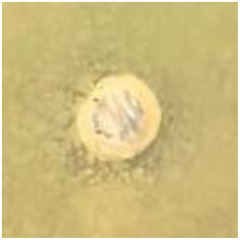

**Table 2:** Minimal inhibitory concentrations (MICs) of selected antimicrobial agents against Gram-positive and Gram-negative bacteria (*n* = 3, SD < ±5.0).

Antimicrobial Agents	MICs (μg/mL)			
	*S. aureus*	*S. epidermidis*	*E. coli*	*P. aeruginosa*
**AgNPs**	4000.0	4000.0	4000.0	1250.0
**Vancomycin**	7.8	7.8	1000.0	1000.0
**LL37**	500.0	500.0	125.0	250.0
**Pexiganan**	31.3	7.8	62.5	31.3
**TTO**	67.1	179.0	33.6	268.5
**CLO**	26.2	26.2	19.7	39.3
**NO**	137.0	182.6	137.0	365.2
